# Paraurethral Endometriosis as a Common Pathology in an Uncommon Location: A Case Report and a Review of the Literature

**DOI:** 10.7759/cureus.35024

**Published:** 2023-02-15

**Authors:** Jawaher Alsahabi, Mohammad Dendini, Eman F Al-Zahrani, Abdulaziz Alosiami, Elham Bamanie

**Affiliations:** 1 Urogynecology and Reconstructive Pelvic Surgery Division, King Abdul-Aziz Medical City Ministry of National Guard Health Affairs, Riyadh, SAU; 2 Obstetrics and Gynecology, King Abdullah International Medical Research Center, Riyadh, SAU; 3 Obstetrics and Gynecology, King Saud Bin Abdulaziz University for Health Sciences College of Medicine, Riyadh, SAU; 4 Radiology, King Abdul-Aziz Medical City Ministry of National Guard Health Affairs, Riyadh, SAU; 5 Radiology, King Abdullah International Medical Research Center, Riyadh, SAU; 6 Radiology, King Saud Bin Abdulaziz University for Health Sciences College of Medicine, Riyadh, SAU

**Keywords:** urinary tract endometriosis, paraurethral endometriosis, vaginal endometriosis, paraurethral cyst, endometriosis

## Abstract

Paraurethral endometriosis is an extremely rare condition. To the best of our knowledge, only seven cases with details on variable risk factors have been reported in the English literature. Herein, we present the case of a third nulliparous patient described in the literature at the time of diagnosis. A 30-year-old woman presented with mild urinary symptoms. A well-defined 2.3 cm paraurethral cystic lesion was found on clinical examination, and MRI findings were suggestive of hemorrhagic content, with no evidence of pelvic endometriosis. Complete surgical excision was performed, and the patient's symptoms improved. The patient experienced no recurrence for 10 months postoperatively. The histopathological findings were suggestive of endometriosis. These findings might indicate that embryonic remnants are possible causes of the pathogenesis of paraurethral endometriosis.

## Introduction

Endometriosis occurs when the endometrial glands and stroma are located outside the uterine cavity [1]. Approximately 95% of typical endometriosis cases occur within the pelvis [2]. After the gastrointestinal tract, the second most common location of extrapelvic endometriosis is the urinary system [3-5]. Since many cases are asymptomatic, the prevalence of urinary endometriosis, which is estimated to be approximately 0.3-12% of all cases of endometriosis [3, 6], remains unclear [4].

Urinary tract endometriosis is most commonly found in the bladder and ureters, accounting for 80-90% of all cases of urinary endometriosis. However, cases of renal and urethral endometriosis are extremely rare [3]. Urinary tract endometriosis is usually diagnosed in women in their 30s or 40s. Additionally, 50% of cases had a history of previous pelvic surgery [3]. The pathogenesis of urinary tract endometriosis has not been yet clearly understood. The general hypotheses on the origins of endometriosis comprise embryonic, migration, transplantation, and iatrogenic remnants theory [6].

To the best of our knowledge, there are seven case reports describing paraurethral endometriosis in the English literature. Six of these seven cases are similar to ours, wherein paraurethral endometriosis was an isolated presentation of endometriosis. Vaginal endometriosis is extremely rare [7], with an incidence of 0.02% in women diagnosed with the symptomatic condition [2]. Moreover, The urethra was involved in 2% of women with urinary tract endometriosis [4]. Urethral endometriosis is usually noted and described as a direct continuation of bladder endometriosis [5]. In one case report, an endometriosis lesion was present in a urethral diverticulum [5].

Herein, we present the case of a 30-year-old woman in which a paraurethral cyst was detected while introducing a Foley catheter during her first delivery, after that the patient presented with mild urinary symptoms, and magnetic resonance imaging (MRI) was the screening modality we used in the patient who eventually underwent surgical excision.

## Case presentation

**Figure 1:**
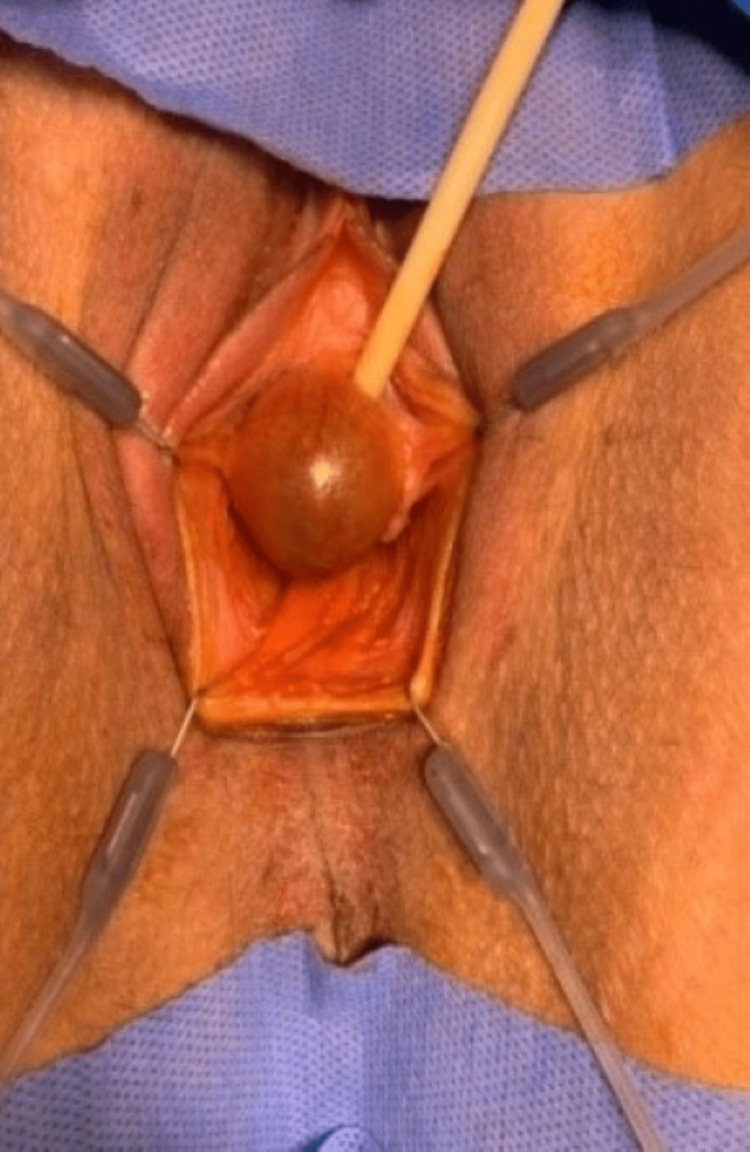
Paraurethral cyst with dark contents

The patient reported in this paper consented for the publication of her case and the accompanying pictures in accordance with the CARE guidelines. A 30-year-old nulliparous woman was found to have a paraurethral cyst while a Foley catheter was inserted during a cesarean section. Four years after delivery, the patient considered planning a new pregnancy and presented to our institution with a deviated urine stream, increased urine frequency, and nocturia. The patient's menstrual cycle was regular, without dysmenorrhea, dyspareunia, or pelvic pain. She had been on oral contraceptives for a period of time. Upon examination, the urethra was pushed to the left side by a distal paraurethral cystic lesion (2.5 cm x 2.5 cm). The cyst was tense, thin-walled, and contained dark bluish contents (Figure 1).

**Figure 2:**
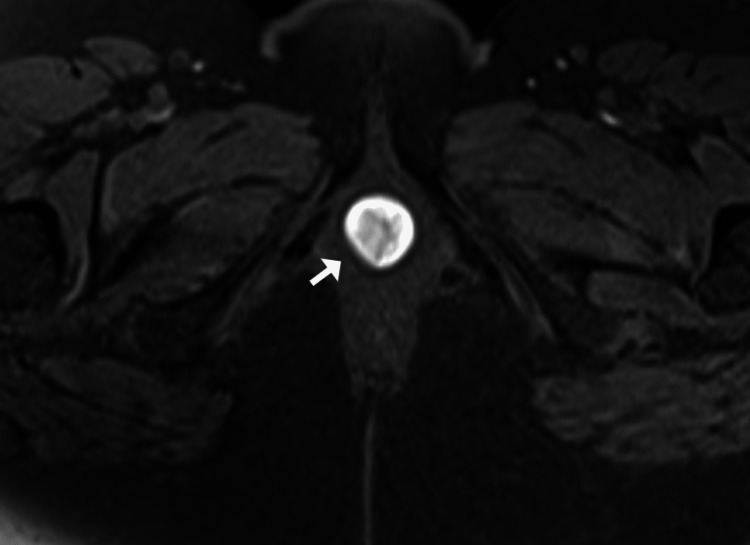
Axial T1 fat-saturated images showing T1 bright paraurethral cyst suggesting hemorrhagic contents

**Figure 3:**
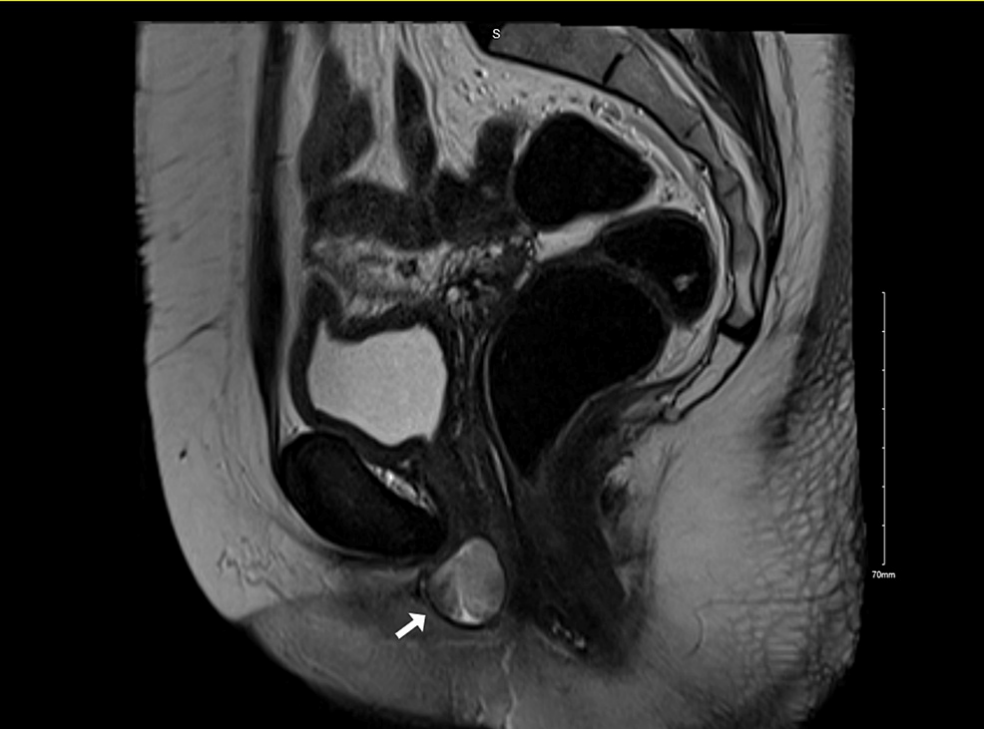
Sagittal T2 image shows that the paraurethral cyst is intermediate on T2 weighted images suggestive of hemorrhagic fluid with shading effect, which indicates recurrent chronic bleeding

A well-defined cystic lesion was detected on MRI in the periurethral region, measuring 2.2 x 2.3 x 1.8 cm (Figures 2, 3). Axial T1 fat-saturated images revealed T1 bright paraurethral cyst suggesting hemorrhagic content. A sagittal T2 image shows that the paraurethral cyst is intermediate on T2 weighted images suggestive of hemorrhagic fluid with the shading effect, which indicates recurrent chronic bleeding.

**Figure 4:**
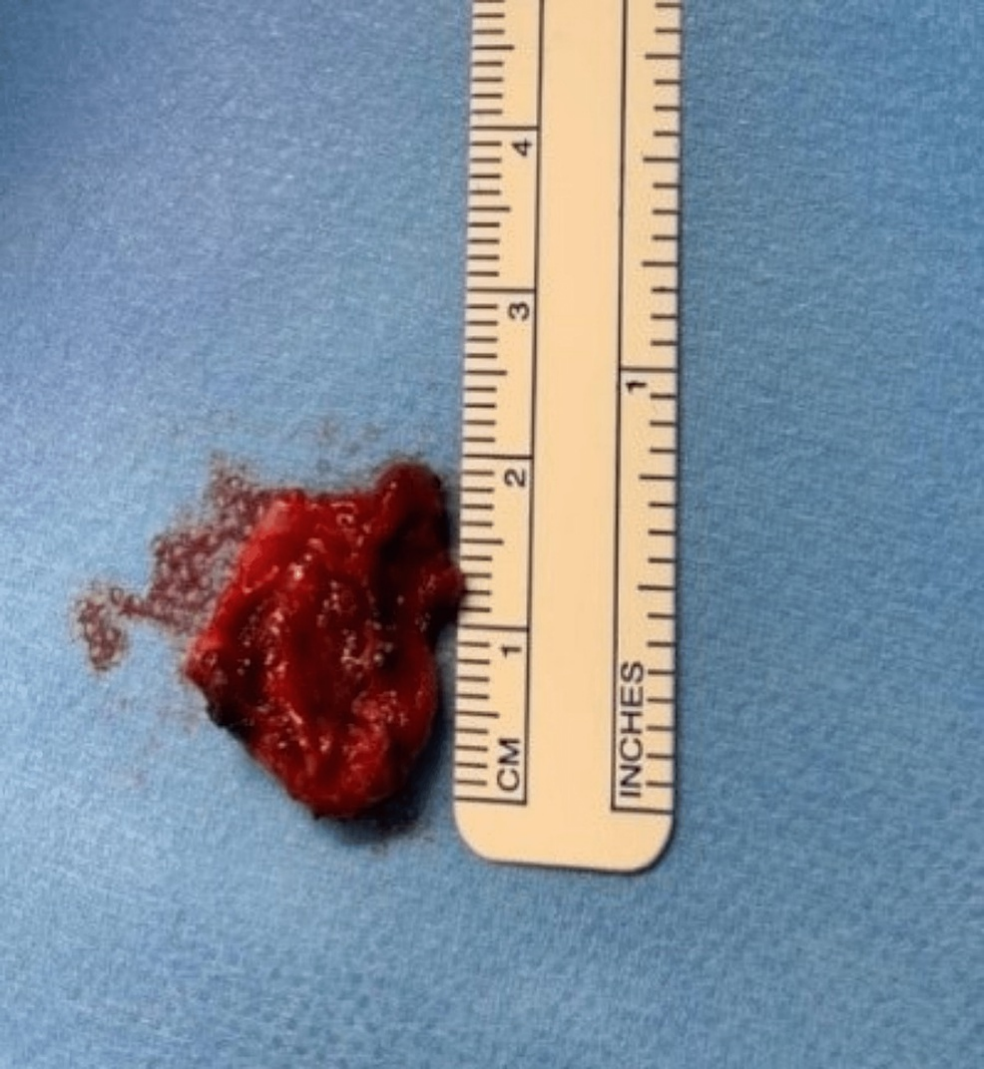
Excised specimen

**Figure 5:**
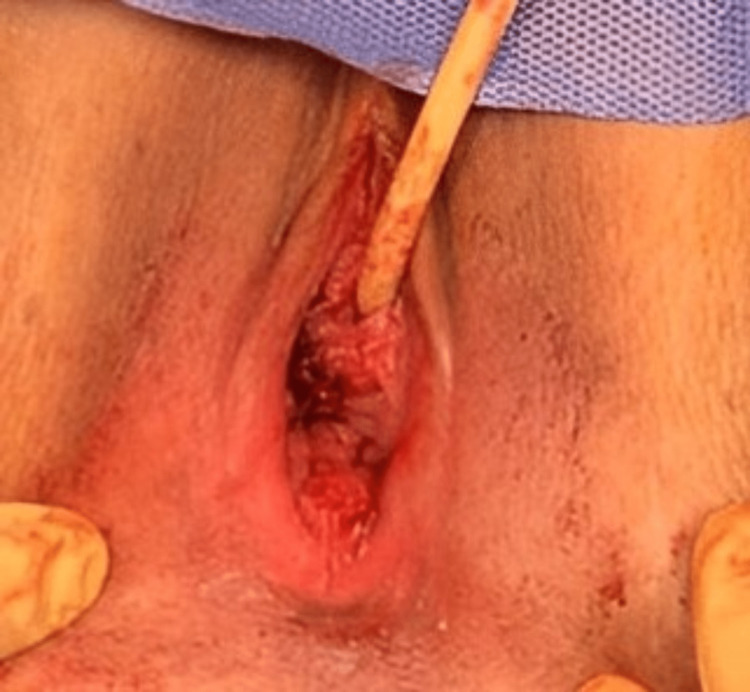
Urethra post resection

The patient underwent cystourethroscopy, which revealed a normal urethra and bladder mucosa with no connection to the cyst. A Foley catheter was introduced, and the area around the cyst wall was infiltrated with epinephrine. Electrosurgery and sharp dissection were performed to excise the cyst (Figure 4). The dead space was closed by interrupted simple stitches, followed by the closure of the external vaginal mucosal epithelium with plication using interrupted simple stitches. The urethra appeared in the normal position after the cyst resection (Figure 5). All suture materials were delayed absorbable. The patient tolerated the procedure very well, without complications, and was hospitalized for two days post-surgery and discharged with an acceptable post-residual volume. During the follow-up period, the final histology diagnosis revealed endometriosis.

**Figure 6:**
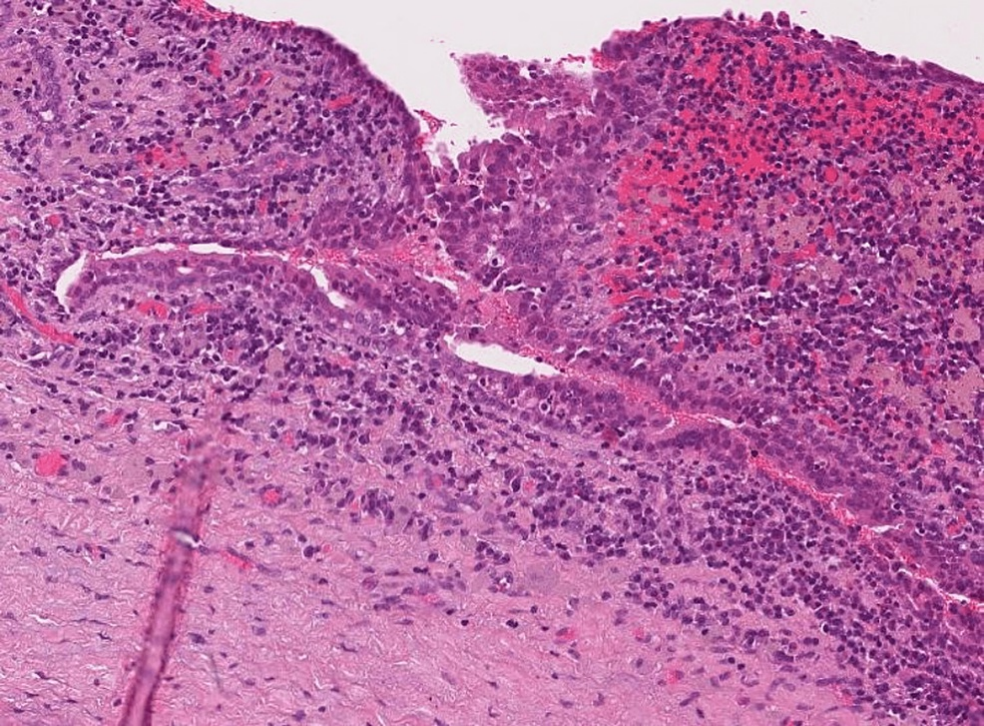
Histopathology of excised cyst

**Figure 7:**
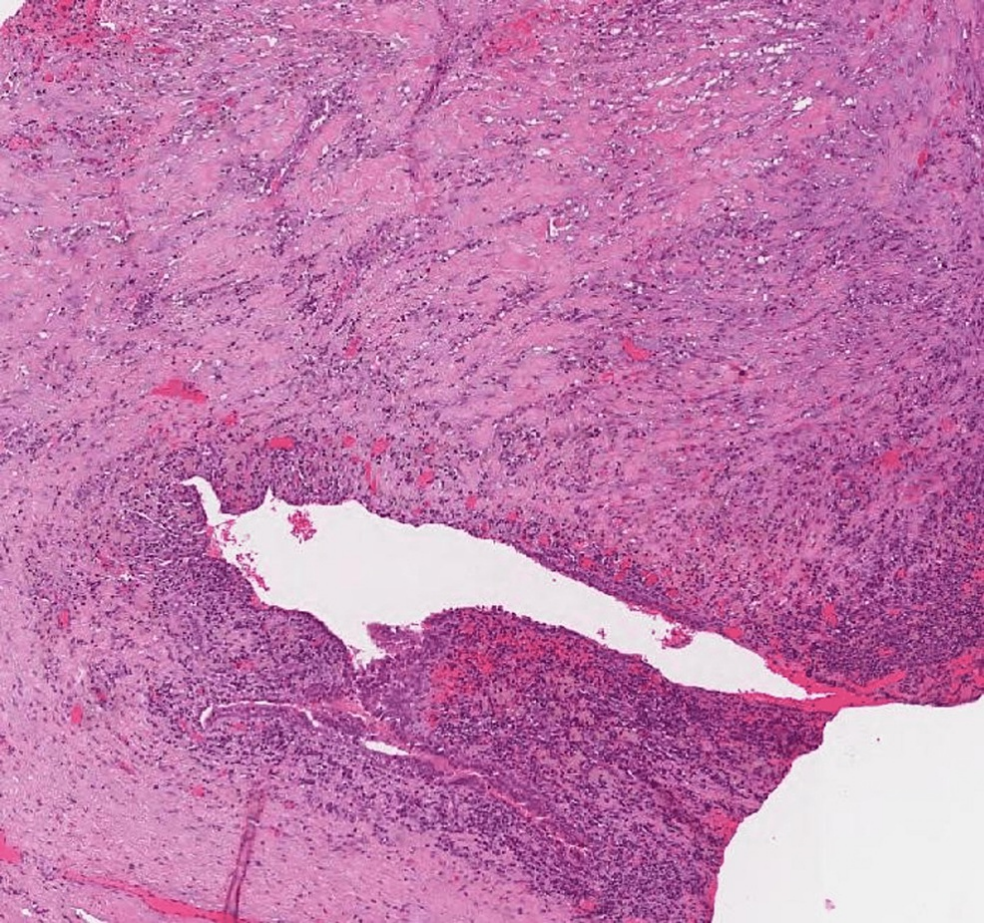
Histopathology of excised cyst

**Figure 8:**
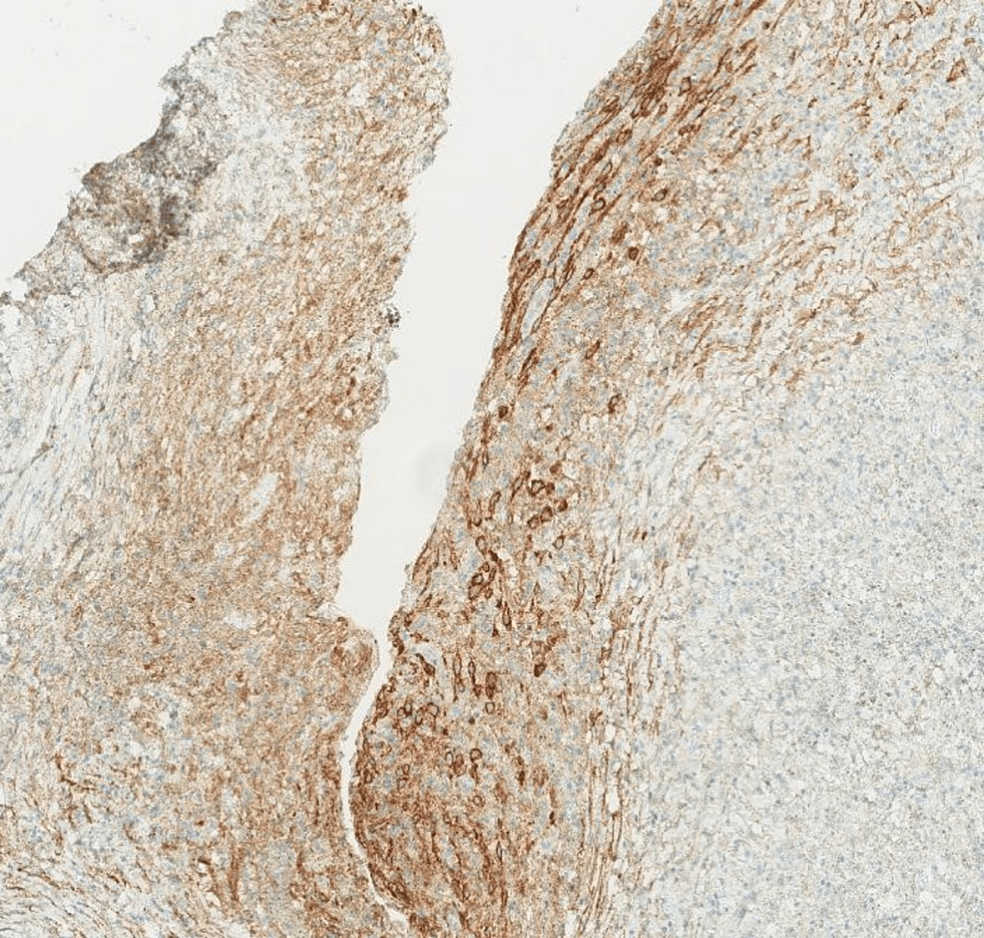
The immunohistochemistry results show CD10 positivity (strong and diffuse cytoplasmic staining) of the stroma, confirming the endometrial differentiation

The Hematoxylin and Eosin microscopic examination shows Müllerian type surface epithelium, conspicuous spindled endometrial stroma with typical capillaries (Figures 6, 7, 8). There was classical subepithelial hemorrhage. There is a focal myxoid change present that is commonly seen in endometriosis.

The patient was followed up for 10 months post-surgery without recurrence of the cyst or recurrence of symptoms.

## Discussion

Since endometriotic paraurethral cyst is a very rare entity with unclear pathology, we searched the literature for similar cases: only seven cases were reported, of which the first was published in 1978.

Endometriosis affects 5-15% of premenopausal women [6]. Urinary tract endometriosis is classified as primary or secondary. Primary urinary tract endometriosis is spontaneously found in the urinary tract and was reported in approximately 11% of women with deep infiltrating endometriosis. In contrast, secondary endometriosis is normally observed after pelvic surgery [6].

Embryonic, migration, transplantation, and iatrogenic theories have been proposed for the pathogenesis of endometriosis [8]. Considering cases similar to ours, wherein the patient had no previous surgery or diagnosis of endometriosis at any other site, we suggest that embryonic remnants of the Müllerian ducts might have been the appropriate theory [5]. Christensen et al. proposed the hypothesis that vaginal endometriosis may occur during vaginal delivery via direct spreading and implantation of endometrial spots [8]. However, in all the published cases that we reviewed, three women were multiparous, two did not report parity, and two were nulliparous similar to our case. This may support the embryonic remnant theory, rather than direct spreading and implantation of endometrial spots [8].

A diagnosis can be confirmed using transvaginal ultrasound and MRI, which can also help in evaluating extension of the disease and help with surgical planning [5, 7]. Transvaginal ultrasound is recommended as the first-line imaging modality in patients with suspected endometriosis. However, its diagnostic accuracy has been shown to be operator dependent [7]. MRI is the gold standard for identifying deeply infiltrating endometriosis and ruling out coexisting endometriosis at other pelvic sites. Computed tomography has a limited role in diagnosing endometriosis [5, 7].

Vaginal endometriosis cysts are unilocular thin-walled cysts that are usually filled with hemorrhagic debris and always demonstrate characteristic T1-hyperintensity and relative T2-hypointensity [7]. Other reported modalities used for diagnosis include contrast radiology (double-balloon positive pressure urethrography or voiding cystourethrography) and cystoscopy [2].

Differential diagnoses should include cysts, such as urethral diverticulum, Gartner's duct cysts, Skene's gland cysts, inclusion cysts, or masses such as condyloma, leiomyoma, fibroepithelial polyp, hemangioma, urethral caruncle, cystocele, and malignancy [2].

Vaginal endometriosis is usually encountered as a sequela of deep infiltrating endometriosis located in the rectovaginal septum or posterior vaginal fornix. However, endometriosis can also be diagnosed in the distal parts of the vagina, which accounts for less than 1% of extrapelvic cases. [8].

All reported cases in our review were treated with complete surgical excision, with successful symptom control and no reported recurrence. Incomplete resection of lesions leads to an increased risk of recurrence and poor symptom control [9].

The therapeutic strategy includes medical and surgical options. However, medical therapy is limited in patients with urinary tract endometriosis, particularly in those with extensive pelvic disease. Therefore, medical hormone suppression should be considered as an adjuvant therapy in patients with concomitant pelvic endometriosis undergoing surgery [3]. A summary of the studies found in the literature is included in Table 1.

**Table 1:** Summary of the literature review of the reported cases of paraurethral endometriosis cyst DIE - deep infiltrating endometriosis, IVP - intravenous pyelogram

Method	Presence of endometrioses elsewhere	Presentation	Proposed risk factors	Size	Location	Parity	Age (years)	Country	Author (year)
MRI	Pelvic peritoneal endometriosis	Stress incontinence, decreased urine flow, and nocturia	Repeated urogynecology surgery	2 cm	Lower third of the anterior vaginal wall close to the urethra	3	45	Denmark	Christensen et al. (2021) [8]
	Extensive pelvic DIE	Chronic pelvic pain that worsened with menstruation, dyspareunia, and dysuria	Known extensive endometriosis	2 cm	Suburethral bluish cystic-appearing lesion	N/A	31	USA	Youssef et al. (2021) [10]
MRI	Not known	Inability to “get a good seal” with a menstrual cup and dyspareunia	None	2 cm	Mid anterior of the vagina at the level of the bladder neck	nulliparous	23	USA	Dilday et al. (2020) [7]
US MRI	Not known	Vaginal lump, discomfort with walking, dyspareunia, and increased urinary frequency	None	3 cm	Directly below the urethral meatus	2	43	Australia	Nelson (2018) [2]
"not specified"	Adenomyosis	Left lower abdominal pain, dyspareunia, dysmenorrhea, increased urinary frequency, and frequent urinary tract infections	None	1.5 cm	Left of the mid urethra	2	35		Chowdhry et al. (2004) [11]
US double balloon cystography	Not known	Painful suburethral mass, dyspareunia, and voiding difficulty	None	3.7 cm	Suburethral	Nulliparous	27	Taiwan	Wu et al. (2003) [12]
CUG urethroscopy IVP X-ray positive pressure urethrogram	Not known	Painful swelling, dyspareunia and dysuria, post voiding leak	None	N/A	Distal one-third of the urethra	N/A	24	USA	Palagiri (1978) [13]

## Conclusions

There is very limited data, of which only a few cases of paraurethral cysts were reported. The two previously reported cases were similar to our case of a paraurethral endometriosis cyst in a nulliparous woman. Even if pelvic endometriosis has not been diagnosed, a high level of clinical suspicion in such cases would lead to appropriate diagnosis and management. Complete surgical excision was the primary treatment in all cases. Additional cases and high-quality research are required to accumulate knowledge on this topic.
